# Insights into the Effects of the Dental Stem Cell Secretome on Nerve Regeneration: Towards Cell-Free Treatment

**DOI:** 10.1155/2019/4596150

**Published:** 2019-10-24

**Authors:** Rohaina Che Man, Nadiah Sulaiman, Ruszymah Bt Hj Idrus, Shahrul Hisham Zainal Ariffin, Rohaya Megat Abdul Wahab, Muhammad Dain Yazid

**Affiliations:** ^1^Tissue Engineering Centre, Universiti Kebangsaan Malaysia Medical Centre, Jalan Yaacob Latif, 56000 Cheras, Kuala Lumpur, Malaysia; ^2^Department of Physiology, Faculty of Medicine, Universiti Kebangsaan Malaysia Medical Centre, Jalan Yaacob Latif, 56000 Cheras, Kuala Lumpur, Malaysia; ^3^Malaysia Genome Institute (MGI), National Institute of Biotechnology Malaysia (NIBM), Jalan Bangi, 43000 Bangi, Selangor, Malaysia; ^4^Faculty of Science and Technology, Universiti Kebangsaan Malaysia, Bangi, Selangor 43600, Malaysia; ^5^Department of Orthodontic, Faculty of Dentistry, Universiti Kebangsaan Malaysia, Jalan Raja Muda Abdul Aziz, 50300 Kuala Lumpur, Malaysia

## Abstract

Cell-free treatment is emerging as an alternative to cell delivery to promote endogenous regeneration using cell-derived factors. The purpose of this article was to systematically review studies of the effects of the dental stem cell secretome on nerve regeneration. PubMed and Scopus databases were used where searched and related studies were selected. The primary search identified 36 articles with the utilized keywords; however, only 13 articles met the defined inclusion criteria. Eight out of thirteen articles included *in vivo* and *in vitro* studies. We classified the dental stem cell-derived secretome with its nerve regeneration potential. All studies demonstrated that dental stem cell-derived factors promote neurotrophic effects that can mechanistically stimulate nerve regeneration in neurodegenerative diseases and nerve injury. This data collection will enable researchers to gather information to create a precise formulation for future prescribed treatments.

## 1. Introduction

### 1.1. Neurodegenerative Diseases, Risk Factors, and Current Pharmacotherapy

Neurons are the cells composing the nervous system, including the spinal cord and brain. Neurodegenerative diseases can result when damaged neurons cannot be replaced or reproduced by the body [1, 2]. This condition is normally related to neuronal structure damage and function failures, factors that cause neuronal death [3]. The significant process of neurodegeneration results in myriad neurodegenerative diseases, including Parkinson's disease (PD), Alzheimer's disease (AD), Huntington's disease (HD), dementia, and spinal muscular atrophy [4, 5]. Unfortunately, continuous nerve deterioration, which predominantly affects human brain and spinal cord, is incurable and contributes to movement and mental function problems [6].

The significantly high incidence of neurodegenerative diseases has attracted increased attention in the past decades. PD commonly affects the central nervous system (CNS) and causes abnormal movement that is characterized by progressive loss of muscle control [7]. The projected prevalence of PD in the US will increase substantially. It is more frequent in men compared to women, with an estimated prevalence of 572 individuals per 100,000 among those aged ≥45 years. These numbers are estimated to increase from 930,000 to 1,238,000 in 10 years, as projected by the US Census Bureau [8], and this elevation represents a considerable medical problem and social burden. AD is one of the most common diseases that leads to dementia and depreciation of cognitive function. Approximately 1 million new AD cases are expected to develop every year, with estimated prevalence ranging from 11-16 million [9]. HD is characterized by abnormal cognitive, emotional, and behavioral functions [10]. Intriguingly, the estimated HD prevalence varies up to tenfold depending on the world region. The prevalence in Australia, North America, and Western Europe had escalated over the past 50 years, whereas lower HD rates are reported for Asian populations [11].

Although the etiology for neurodegenerative diseases remains elusive, many recent studies suggest prominent risk factors. Most of the known risk factors include environmental pollutants [12], ageing [13], oxidative stress [14], chemical exposure [15], and infection [16]. There are myriad pharmacotherapies that were investigated to treat the diseases. Acetylcholinesterase inhibitors and N-methyl-d-aspartate (NMDA) receptor agonist both offer a good therapy choice, especially for AD [17]. In the clinical setting, this particular therapy has attracted significant research interest in order to evaluate the efficacy of pharmacotherapy for AD. A recent study by Manenti et al. [18] revealed significant improvements in motor abilities and a reduction of depressive symptoms in PD patients through anodal transcranial direct current stimulation applied over the dorsolateral prefrontal cortex combined with physical therapy.

In recent decades, researchers have made numerous efforts to elucidate the mechanism(s) of neurodegenerative diseases and possible pharmacotherapies that can help to decelerate and prevent these diseases from worsening. The current medical treatment tends to be palliative rather than curative. Unfortunately, none of them significantly halts the underlying pathology. This review article will expound upon the core value of dental stem cells (DSCs), with special emphasis on dental pulp stem cells (DPSCs) and stem cells from human exfoliated deciduous teeth (SHEDs), and the role of their paracrine factors for potential future applications in neurodegenerative disease therapies.

### 1.2. DSC Secretome

**Figure 1:**
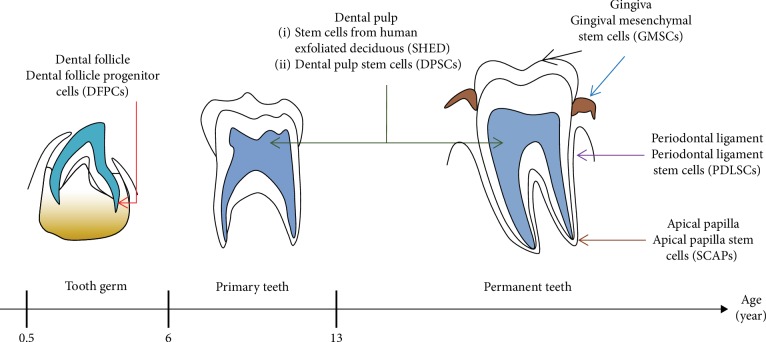
Tooth developmental stages with the anatomical localization of the difference dental-derived stem cells in a tooth germ, primary teeth, and permanent teeth. Different subpopulations of DSCs can be categorized according to their tissue of origin. Modified from [74].

DSCs can be isolated from various dental soft tissue. They can be divided into several categories according to the origin [19].  Figure 1 shows the anatomical localization of the different DSCs starting from tooth germ, primary teeth, and permanent teeth. Dental follicle progenitor cells (DFPCs) can be isolated from dental follicle tissue of the tooth germ as early as 6 months old. SHEDs can be isolated from primary teeth at 6 years old. Various DSC populations can be isolated from permanent teeth, including DPSCs, periodontal ligament stem cells (PDLSCs), apical papilla stem cells (SCAPs), and gingival mesenchymal stem cells (GMSCs), which can be isolated from dental pulp, periodontal ligament, apical papilla, and gingiva, respectively (Figure 1) [19, 20].

PDLSCs have vital stem cell properties, including high multipotency, great ability for self-renewal, and the ability to express most stem cell markers, i.e., CD166, STRO-1, and CD105 [21]. Hence, the role of PDLSCs could be important in preserving periodontium as well as periodontal regenerative procedures. SCAPs are distinctive stem cells that are promising for endogenous tissue regeneration [22], pulp/dentin regeneration, and bioroot engineering [23]. They are a very unique cell population of postnatal stem cells that are different from DPSCs, in terms of cell motility and migration [24]. This activity will allow the cells to develop into a complex tissue and organ during regeneration; thus, it can be considered as one of the alternative cell resources for neurodegenerative disease therapies. SCAPs have less cellular and vascular components than those in the pulp, and they have osteogenic and dentinogenic potential (because they are mesenchymal stem cells [MSCs]) [25]. A recent study from Simonovic and coworkers [26] demonstrated that SCAPs cultured in neurogenic induction medium supplemented with graphene dispersion and water-soluble single-walled carbon nanotubes exhibit an elevated capacity to differentiate into neural lineage cells. DFSCs come from highly fibrous tissues that are usually extracted and discarded in dental surgery. DFSCs can be cultivated under various culture conditions and thus could be used in tissue engineering and regenerative therapy applications, including neural differentiation [27] and periodontal [20] and tooth root regeneration [28].

SHEDs, PDLSCs, and DFSCs can be obtained from 6- to 12-year-old individuals [29]. SHEDs possess a great proliferative capacity and the ability to differentiate into adipocytes, neurons, and odontoblast-like cells. They are easily obtained (with minimal or no trauma) due to simple, convenient, and relatively noninvasive techniques [30]. The DPSCs play an important role in tooth homeostasis and remain active throughout life to generate odontoblasts for dentine repair. *In vivo*, stem cells differentiate according to their specific functions under the action of signaling molecules in a microenvironment called the “stem cell niche.” This phenomenon reflects the stem cell native microenvironment, whereby it is thought to preserve the properties and functions of stem cells and monitor differentiation.

DPSCs that are derived from the embryonic cranial neural crest are one of the distinctive types of ecto-MSCs. In the tooth, the DPSCs is located at certain anatomical locations that forming stem cell niches. This niche microenvironment modulates the DPSC populations to promote tissue repair and regeneration [31]. Many signaling molecules in the niche are essential to maintain the stem cell activities, which also have a capacity to regulate cell proliferation and differentiation. DSPCs can differentiate into neural cells to ameliorate nervous system damages [32–34]. They can also differentiate into nonneural cells, including the cartilage [35], bone [36], liver [37], corneal stroma [38], retina [39], and tendon-like tissue [40]. DPSCs can be isolated from dental pulp of third molar teeth without invasive surgery; they are easily cultivated *in vitro* and expanded for research use. Stem cells harvested from the other tooth regions that involve the infant's exfoliated deciduous immature teeth are known as SHEDs [41]. Many recent updates revealed that DPSCs possess a good proliferative capacity and are multipotential. They can differentiate into neurogenic [42], osteogenic [43], odontogenic [44], and chondrogenic [45] lineages. DPSCs express MSC-like markers (e.g., STRO-1, CD29, CD105, and CD90) [46] and neural stem cell-like markers (e.g., nestin and glial fibrillary acidic protein) [47]; this expression pattern signifies their self-renewal and multipotency capacity. Interestingly, pluripotent stem cell markers such as Oct4, Nanog, Sox, and Klf4 are also regulated by DPSCs. Furthermore, DPSCs have more potent neurogenicity properties and immunosuppressive activities compared to bone marrow mesenchymal stem cells (BMSCs). The abovementioned DPSCs properties make them a strong potential candidate to cure ischemic neurodegenerative disorders [48]. Thus, they could play a significant role in treating neurodegenerative conditions in the human body.

Recent studies reported that secretomes, or conditioned media (CM) acquired from a wide variety of stem cells, can efficiently impede organ damage and ischemic disease. The secretome represents the entire array of proteins and factors that are secreted by a cell into the extracellular space; it constitutes approximately 30% of the entire proteome in an organism. The secretome contains growth factors, cytokines, chemokines, antibodies, receptors, adhesion molecules, hormones, enzymes, toxins, peptides, proteinases, and antimicrobial peptides. Most of these proteins are actively involved in various biological processes that typically comprise cell attachment [49], proliferation, migration, and differentiation [50], intracellular communication [51], immune response [52], cell survival, and cell defense [53]. All these metabolic and homeostatic processes are essential for the continuity and transformation of life. Previous investigations postulated that DPSC-CM greatly contributes to regenerative therapy, mainly in the CNS [54] and retinal disorders [55]. DPSC-CM offers a therapeutic effect that may implicate a diverse pathway, particularly through the intervening paracrine mechanisms that activate repairing activities. Thus, the repertoire of DPSC-secreted trophic factors might be a significant contributor because it is necessary for neural regeneration.

DSC-CM is useful in enhancing long-term neuronal regeneration in spinal cord injury. DPSC-CM significantly improves cognitive function in a mouse model of AD, specifically by converting the proinflammatory conditions to an anti-inflammatory state. The multifaceted activities offered by SHED-CM may provide neuroprotective effects and could be considered as a potential treatment for the neurodegenerative disorders [56]. A recent study by Yamamoto et al. [57] revealed reduced apoptosis and active proliferation of Schwann cells in the DPSC transplant as opposed to the control conduits. Further *in vitro* analysis demonstrated that DPSCs promote axon regeneration and stimulate angiogenesis through trophic functions. A study reported by Tsuruta and coworkers [58] showed that systemic SHED-CM administration in a rat subjected to superior laryngeal nerve (SLN) injury successfully improves SLN functional recovery, namely by significantly enhancing axonal regeneration by transforming macrophages to the anti-inflammatory M2 phenotype. It also contributes to angiogenesis at the injured site. Thus, SHED-CM administration may represent an alternative therapeutic option for SLN injury. Considering this evidence, an excellent and noninvasive acellular tool like DPSC-CM and SHED-CM should be further explored for future use in regenerative therapy. In this concise review, we focused on the recent findings using DPSC-CM and SHED-CM for nerve repair, neuroprotection, and neuroregeneration in neurodegenerative diseases and nerve injury.

## 2. Methods

### 2.1. Search Strategy

This review was systematically conducted by screening all published articles on the effects of the DPSC secretome on nerve regeneration. Two databases were comprehensively used to search for related study (Scopus and PubMed). The keywords used were the combination of dental pulp stem cell secretome OR DPSC secretome OR dental stem cell secretome OR dental pulp stem cell conditioned medium OR DPSC conditioned medium OR dental stem cell conditioned medium AND nerve regeneration OR nerve development OR nerve repair.

### 2.2. Selection Criteria

Studies published in English from 2000 to 2019 were considered for inclusion. Only articles that provided the full paper were selected. The titles and abstracts were carefully screened to meet the related topic of interest. Primary studies related to the DPSC secretome production, neurotrophic effects, and nerve regeneration potential were included. Only research articles were selected. Review articles, news articles, letters, editorials, and case studies were excluded from the search.

### 2.3. Data Extraction and Management

All data were extracted from selected articles by two reviewers. The selected papers underwent three screening phases prior to inclusion. The title was first screened for relevance to the topic of interest. Then, the abstracts were carefully screened and unrelated studies were excluded. Lastly, all duplicates were removed. The data were summarized in a table as follows: (1) authors, (2) type of secretome, (3) donor age/condition, (4) type of nerves/disease or cells studied, (5) methodology, (6) passage number/type of medium/period of culture for secretome collection, (7) results, and (8) conclusions.

## 3. Results

### 3.1. Search Results

**Figure 2:**
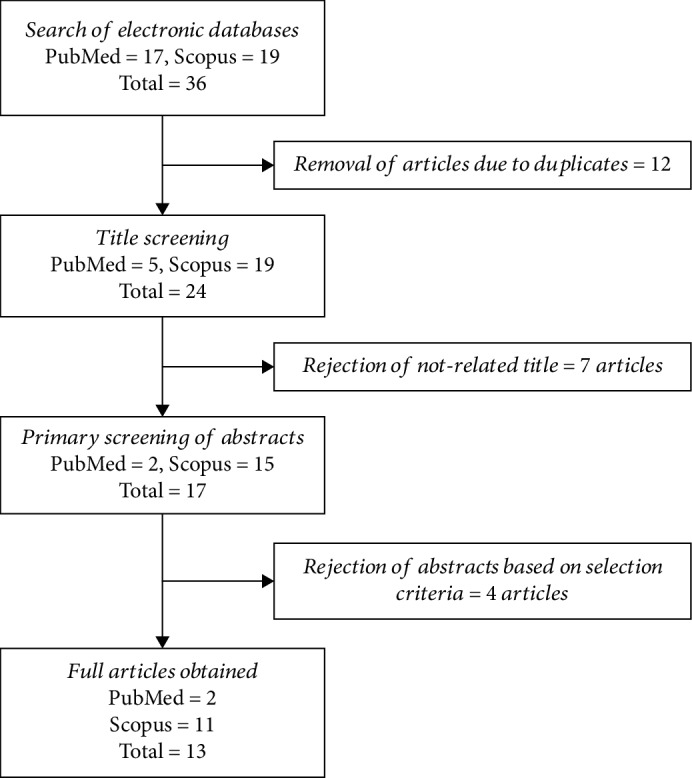
Flow chart of the article selection process from PubMed and Scopus and databases.

Two reviewers independently assessed the articles according to the defined inclusion and exclusion criteria. This procedure was performed to minimize bias while selecting articles. At the end of the selection session, a joint discussion was conducted to achieve consensus when differences emerged during the assessment. The primary searches that used the combination of keywords (Section 2.1) only identified 36 articles: 17 from PubMed and 19 from Scopus. Twelve duplicate articles were excluded by title sorting prior to full paper search. After title screening, seven articles were rejected based on the inclusion criteria; these articles were not related to nerve regeneration. Finally, a total of 13 studies were selected for data extraction in this review. The flow chart of the selection process is shown in Figure 2.

### 3.2. Study Characteristics

The database search provided 13 articles related to DSCs, secretome, CM, nerve regeneration, and neurogenesis. From these articles, various types of dental tissue sources, i.e., SHEDs and adult dental tissue, were used for potential secretome collection/production. SHEDs were extracted from donors aged 6 to 12 years, while adult DSCs came from donors aged 13 to 29 years. The secretome was derived from cells at passage 3 to 9 after 24- to 48-hour culture in serum-free Dulbecco's modified Eagle's medium (DMEM) or minimum essential medium, Eagle alpha modification (*α*MEM). One study reported the secretome content in detail, with a focus on a set of M2 macrophage inducers (monocyte chemoattractant protein-1 [MCP-1] and secreted ectodomain of sialic acid-binding Ig-like lectin-9 [sSiglec-9]) in the SHED-CM [59]. The remaining articles directly tested the secretome *in vitro* and *in vivo* (without prior characterization) to observe their potentiality for neurodifferentiation. One study profiled the secretome from SCAPs [20]. Eight out of thirteen studies conducted *in vivo* research: 7 in rats and 1 in mice. One study reported the therapeutic effects of intravenous administration of the secretome in a rat model [27]. Most studies compared the secretome derived from BMSCs and dental DSCs [20, 27]. All of the studies concluded that the secretome has neurotropic effects on specific nerve repair and regeneration. A summary of the studies is provided in Table 1.

## 4. Discussion

In the past decade, numerous studies reported that MSCs, especially DSCs, can regenerate injured nerves by promoting axonal regeneration and myelin sheath formation. DSCs share a common origin with peripheral nerves and express neuronal markers [27, 32, 33]. Engrafted DSCs alone are susceptible to ischemic attack. However, with appropriate paracrine factors, cells adjacent and distal to the injury site can be influenced to create a unique microenvironment for the stem cells to be functional.

Generally, the secretome includes molecules secreted from cells into the extracellular space; it includes free nucleic acids and soluble proteins and lipids, along with extracellular vesicles (EVs), i.e., microvesicles (MVs) and exosomes that act as intercellular mediators to carry those entities. This broad range of bioactive soluble factors is antiapoptotic, antifibrotic, and anti-inflammatory, and they contain angiogenic regulators, chemoattractive factors, neurotrophic factors (NTFs), and immunomodulators. Stem cells release these molecules through classical and nonclassical secretion mechanisms, including protein translocation, exocytosis, and exosome encapsulation as means of cell-to-cell communication [35].

DPSCs, in particular, secrete various growth factors (GFs) and cytokines. Previous studies revealed high expression levels of transforming growth factor (TGF) and NTFs [60–62]. The NTFs, including nerve growth factor (NGF), glial cell-derived neurotrophic factor (GDNF), neurotrophin-3 (NT-3), brain-derived neurotrophic factor (BDNF), ciliary neurotrophic factor (CNTF), vascular endothelial growth factor (VEGF), and hepatocyte growth factor (HGF), are initially necessary for the innervation of dental tissues. Intriguingly, these factors are also vital for the restoration of neural tissues [63].

**Figure 3:**
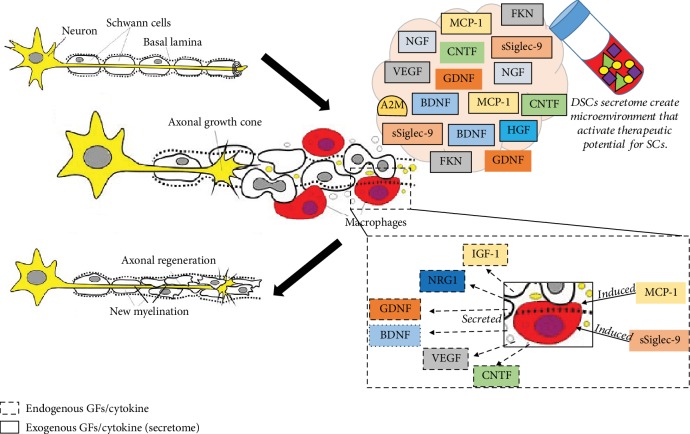
Schematic illustrating the role of DSC secretomes modulating the nerve regeneration in CNS. Alzheimer's disease (AD) responds to the production of *β*-amyloid fibres/plaque which triggers the microglia and astrocytes activation and generation of proinflammatory cytokines. The chronic activation of microglia and astrocytes causes neuron degeneration. Stimulation by GFs and cytokine derived from DSCs secretome such as A2M cytokine is capable of binding to *β*-amyloid fibres/plaque that mediate the clearance and degradation [1] while FKN can execute their phagocytic functions. In addition, Siglec-9 and MCP-1 can switch the M1 to M2 phenotype for nerve regeneration. This would enhance neuronal plasticity and neurogenesis in AD patients.

Neurodegenerative diseases involve brain cell deterioration. In AD, the body starts to produce a protein called amyloid that is deposited as “plaques” in the brain. This phenomenon leads to structural brain changes and consequently prevents the production of neurotransmitters. Previously, alpha-2 macroglobulin (A2M) demonstrated an ability to inhibit amyloid formation [64]. Tachida and colleagues (2015) [60] identified that A2M is the most prominent secreted protein in the DPSC proteome. A2M is a protease inhibitor and cytokine reporter that might play a key role in the neuroinflammatory response to AD pathogenesis [65]. A2M can bind to misfolded and aggregation-prone client proteins; this process can mediate the clearance and degradation of *β*-amyloid deposits in AD patients [66, 67]. Therefore, exogenous A2M derived from the secretome would be expected to at least slow down the progression of brain cell death in AD patients (Figure 3).

Peripheral nerve injury (PNI) is caused by trauma or surgical complications that leads to distal stump demyelination and degradation. Typical symptoms are motor sensory deficits, including weakness, paralysis, and pain. Current treatments, such as direct repair and autologous nerve grafts, are still insufficient. Recently, Tsuruta and colleagues (2018) [58] reported that the DPSC-derived secretome can induce neuronal regeneration. Based on their findings, the authors suggested that the systemic administration of SHED-CM may provide therapeutic benefits in PNI treatment. As previously reported by Sugimura-Wakayama and coworkers (2015) [68], the SHED-CM secretome contains NGF, BDNF, NT-3, CNTF, and GDNF, all of which create a more desirable extracellular microenvironment for peripheral nerve regeneration [34]. They reported that SHED-CM enhances migration and proliferation *in vitro*. It also promotes axonal regeneration and functional recovery in a sciatic nerve defect rat, including enhanced axon growth, angiogenesis, migration, proliferation, and neuron survival. This data indicate that SHED-CM contains factors that can regulate the mobilization of Schwann cells to the target tissue. Thus, it serves as a potential PNI treatment [34].

**Figure 4:**
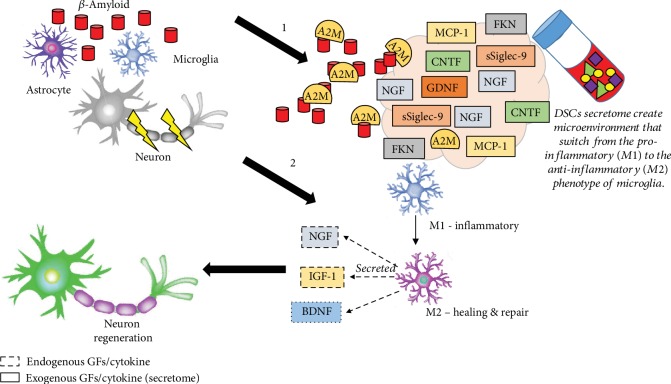
Schematic illustrating the role of DSC secretomes modulating the nerve regeneration in PNS. Axon of the neuron is myelinated by the Schwann cells (SCs). Nerve injury has caused the myelin sheaths and axon degenerated. There are series of macrophage activation by several GFs and cytokine involved to remove the debris of degenerating fibers like A2M cytokine that recognized by specific receptor on macrophages. On the other hand, MCP-1 and sSiglec-9 induced the polarization of M2 macrophage. This has caused MCP-1/sSiglec-9-induced M2 macrophages expressed six factors that are known to affect the functional properties of SCs. The six factors are IGF-1, NRG1, BDNF, CNTF, VEGF and GDNF. These factors promote the proliferation, migration, and differentiation of SCs that can enhance axonal regeneration. Neuron image adopted and modified from Mey et al. [75].

Macrophages involved in distal degeneration can promote the switch from the proinflammatory (M1) to the anti-inflammatory (M2) phenotype. A set of tissue-repairing M2 macrophage inducers, i.e., sSiglec-9 and MCP-1, enhance nerve regeneration [29, 69]. SHED-CM that contains MCP-1 and sSiglec-9 enhances neurite extension of the peripheral nerve, data that suggest these factors can promote the formation of a Schwann cell bridge and axonal extension. The depletion of both MCP-1 and sSiglec-9 in SHED-CM reduces its ability to restore neurological function and to regenerate peripheral nerves [29]. On the other hand, a study by Matsubara and colleagues identified the M2 inducers activate multifaceted endogenous neurorepair mechanisms, effects that restore locomotor function in a rat model of spinal cord injury (SCI). M2 inducers directly convert the proinflammatory conditions prevalent in the damaged CNS to tissue-repairing function by modulating the microglia/macrophage phenotype (Figure 4) [69].

Other than NTFs, El-Moataz and colleagues (2016) [70] revealed that the DPSC secretome contains a high concentration of cytokines, including fractalkine (FKN), which is regulated on activation and normally T cell expressed, and presumably secreted RANTES and FMS-like tyrosine kinase 3 (FLT-3). FKN, also known as chemokine (C-X3-C motif) ligand 1 (CXCL1), promotes microglia survival under neurotoxic conditions and enhances the ability of macrophages and microglia to execute their phagocytic functions. The activation of the phagocytic response is important to clear cellular debris and stress-response pathways to counteract any remaining neurotoxic molecules that caused the initial damage, especially in neurodegenerative diseases (Figure 3) [71]. On the other hand, RANTES, also known as chemokine (C-C motif) ligand 5 (CCL5), aids during acute infection by promoting macrophage infiltration, mobilization, and function at injured sites. FLT-3 is involved in differentiation, proliferation, and survival of dendritic cells. RANTES and FLT3 are both implicated in the processing of pain information in peripheral nociception. However, direct evidence and the possible mechanism of this action is lacking.

Collectively, the secretomes derived from SHEDs and DPSCs demonstrate the most potential for nerve regeneration. Besides having a high proliferation rate, DPSCs also exhibit more growth factors and cytokines compared to other MSCs (e.g., BMSCs) [38]. The DSC-CM contains complex soluble signaling molecules and growth factors that can create a potent odontogenic microenvironment, and the paracrine mechanism of release makes them attractive for use in neuroregeneration [39].

Another aspect to consider is the method used to collect the CM. Cell confluency, passage number, incubation time, growth medium, and induction are crucial aspects to ensure that cells secrete distinct, beneficial proteins. DSCs stimulated by specific GFs, including neuregulin, basic fibroblast growth factor (bFGF), platelet-derived growth factor (PDGF), and forskolin, provide an alternative to Schwann cells to support regeneration after PNI [40, 72]. It is also worthy to compare the common two-dimensional cell culture technique with a three-dimensional culture. Previously, three-dimensional BMSC cultures were reported to produce significantly more secretome [41]. However, it still remains unknown whether the three-dimensional DPSC secretome contains “extra” growth factors and signaling molecules to exert their beneficial effects for better execution of neuroregeneration.

Cell-based therapy limitations led scientists to find a new method that can deliver therapeutic value to the patient. It is well-documented that the DSC secretome contains various growth factors and cytokines along with EVs that act as a stable cargo. EVs can be transported into cells via endocytosis. EVs can also penetrate the blood-brain barrier, a crucial feature given that many drugs may not be able to penetrate it [73]. However, the composition of cargo in EVs depends on cell type and culture and induction conditions. Thus, it is very important to identify the cargo in EVs prior to their use for a specific treatment. Therefore, secretome generation must be properly performed with good manufacturing practice (GMP) to achieve a clinical-grade product prior to its use as a treatment in clinical applications.

## Figures and Tables

**Table 1:** Summary and classification of the 13 articles selected from the database search.

No.	Authors	Type of cell secretome derived	Donor age/condition	Type of nerves/disease/cells studied	Methodology	Passage number/type of medium/period of culture for secretome collection	Results	Conclusion
1	Tsuruta et al. 2018 [58]	Human exfoliated deciduous teeth conditioned medium (SHED-CM)	6-12 years	Superior laryngeal nerve (SLN)-PNS	Rat model: the SLN was exposed bilaterally and injured with a vascular clip (60 g/mm^2^) over a period of 30 min in male Wistar/ST rats weighing 300–330 g (9-10 weeks old). Systemic administration in rats with 1 ml SHED-CM injected into the tail vein for 10 s simultaneously.	Unspecified passage number. Conditioned media were collected after 48 hours cultured in serum-free DMEM	SHED-CM promotes axon regeneration after SLN injury. Many nerve myelinated fibers were identified in the SHED-CM group. The SHED-CM group showed higher fiber densities when compared with the DMEM (-) group. G-ratio showed that the degree of myelination in the SHED-CM group was significantly higher compared to the DMEM (-) group	Administration of SHED-CM improved functional recovery in SLN model.
2	Kolar et al. 2017 [72]	DMSC from human third molar age range 12-25 years: (1) Apical papilla stem cells (SCAP) (2) Dental pulp stem cells (DPSC) (3) Periodontal ligament stem cells (PDLSC)	12-25 years	Sciatic nerve-PNS	*In vitro* model (neurite outgrowth assay): SHSY-5Y neuronal cells were retinoic acid-differentiated (48 h) prior to exposure to conditioned media (CM) derived from all dental MSCs. *In vivo* rat model: 10 mm gap was excised (5 mm below sciatic nerve, 5 mm length of sciatic nerve). Fibrin conduits were inserted to the nerve and sutures at each ends. DMSC populations were stimulated with the following: (1) 200 ng/ml neuregulin1-beta1 (R&D systems) (2) 10 ng/ml basic fibroblast growth factor (Millipore) (3) 5 ng/ml platelet-derived growth factor (Millipore) (4) 14 *μ*M forskolin (Sigma), for two weeks	Conditioned medium were collected from 2-week stimulated DMSCs.	*In vitro*: total neurite outgrowth increased in length when cultured in CM compared to medium-only control. *In vivo*: immunohistochemistry analysis demonstrated that DSCs enhance axon regeneration.	Human SCAP, DPSC, and PDLSC provide an alternative to Schwann cells to support regeneration after peripheral nerve injury and repair.
3	Kano et al. 2016	(i) Human exfoliated deciduous teeth conditioned medium (SHED-CM) (ii) sSinglec-9 or MCP-1 depleted-SHED-CM	6-12 years	Rat facial nerves-PNS	Facial nerve injury was created by removing 5 mm segments of the buccal and marginal branches at sites that were 10 mm distal to the stylomastoid foramen. Atelocollagen sponge impregnated with 20 *μ*l (i) CM, (ii) DMEM, (iii) recombinant human MCP-1, and (iv) recombinant sSiglec-9 was placed in the nerve gap.	Passages 3 to 9 after cultured for 48 hours in serum-free DMEM	SHED-CM depleted both MCP-1 and sSiglec-9 showed lost ability to restore neurological function.	MCP-1 and sSiglec-9 in SHED-CM play important role to regenerate peripheral nerves.
4	Kumar et al. 2016	Human DMSC (1) DPSC secretome (2) DFSC secretome (3) SCAP secretome (4) BMSC secretome (control)	11-25 years (donor undergoing tooth extraction for orthodontic reasons)	*In vitro* neuronal differentiation of IMR-32 preneuroblastic cell line using the secretome	All stem cells were maintained with a-MEM for 48 h prior to secretome collection. Secretome were used for neuronal differentiation of IMR-32 preneuroblastic cell line	Passages 3 to 7 after cultured for 48 hours in serum-free *α*MEM	Higher colony forming efficiency of DMSC secretome as compared to BMSC secretome. Neurite extension assay: neurite length was highest for IMR-32 cells treated with DPSC secretome. Neural gene profiling: IMR-32 induced DMSC secretome showed significant regulation of B-tubulin III and sox-1 expression as compared to IMR-32 induced BMSC secretome. Neural regulatory molecules present in secretome: (1) NGF: no significant difference between DPSC and BMSC secretomes (2) BDNF: higher in DMSC compared to BMSC (3) NTF: higher in DMSC compared to BMSC	DPSC secretome molecules can enhance neural differentiation by increasing the expression of neural genes, preventing apoptosis of neurons, or by maintaining a neuronal fate during differentiation.
5	Yamamoto et al. 2016 [57]	Mobilized dental pulp stem cells (MDPSCs) (human third molar)	18-29 years	MDPSC secretomes were used to clarify its effect on Schwann cell in terms of migration, proliferation, and antiapoptotic analyses-PNS	DPSCs were treated with G-CSF to induce stem cell mobilization by culture in Transwell (upper and lower chambers) for 48 h. The medium was changed into serum-free DMEM at 70% confluence and CMs were collected after 24 hours and concentrated using centrifugal filter	Passage 3 after cultured for 24 hours in serum-free DMEM	MDPSC secretome: (1) Enhanced proliferation of RT4-D6P2T cells (rat Schwann cells) (2) Enhanced the migratory activity of RT4-D6P2T cells (3) Decreased apoptosis of RT4-D6P2T cell	MDPSCs contribute to peripheral nerve regeneration by secretion of neurogenic/angiogenic factors in the close proximity of newly migrated Schwann cells while regulating their apoptosis and proliferation.
6	Yu et al. 2016 [46]	Dental apical papilla (SCAPs) (human third molar)	16-24 years	N/A	SCAPs were seeded at 20,000 cells/cm^2^. When reached 90%, cells were washed and cultured in serum-free medium for 24 hours. Collected secretomes were concentrated.	Passages 3 to 5 after cultured for 24 hours in serum-free *α*MEM	SCAP secretomes contain higher secretion of chemokines and neutrophins than BMSCs.	SCAPs were found to secrete angiogenic, immunomodulatory, antiapoptotic, chemokine, and neuroprotective factors.
7	Matsubara et al. 2015 [69]	Human exfoliated deciduous teeth conditioned medium (SHED-CM)	6-12 years	Spinal cord injury/cerebellar granule neurons (CGN)/bone marrow macrophages (BMMs)-CNS	M2-like bone marrow macrophage induction assay—BMMs were subjected to IHC after supplemented with each CM ELISA—measurement of cytokines in CMs and protein depletion assays Cytokine antibody arrays using laser scanning, cytokines in SHED-CM, BMSC-CM, and serum-free DMEM were detected by 274-human-cytokine array plates Western blot, lectin blot, and coimmunoprecipitation—to detect ED Siglec-9 in CMs, the CMs were concentrated up to 50 times	Passages 3 to 9 after cultured for 48 hours in serum-free DMEM	Treatment with SHEDs or SHED-CM promotes functional recovery after SCI. Rats treated with SHEDs or SHED-CM exhibited less tissue loss & more 5-HT-positive descending raphe spinal axon fibers compared with the control. Effect of CMs on the SCI inflammatory response Treatment with SHED-CM or BMSC-CM similarly suppressed these proinflammatory mediators for 1 week after the injury. Factors in SHED-CM that induce macrophage differentiation 79 proteins expressed in SHED-CM, 28 were known to be involved in neuroregenerative processes. SHED-CM lacking of MCP-1 & ED Siglec-9 fails to induce M2 or to restore locomotor function after SCI. The depletion of IL-6 from SHED-CM (IL-6) had little or no effect.	MSC-derived secreted factors directly convert the proinflammatory conditions prevalent in the damaged CNS to tissue-repairing ones by modulating the microglia/macrophage phenotype.
8	Mita et al. 2015 [56]	(1) Human exfoliated deciduous teeth conditioned medium (SHED-CM) (2) Human bone marrow mesenchymal stem cells (BMSCs-CM) (3) Human skin fibroblast (Fibros-CM)	Unspecified	Alzheimer's disease/primary neurons-CNS	All three cells were cultured in SF DMEM. CM collected after 48 h of culture. 50 *μ*l of each CM administered to the ICR mice (9 weeks old) intranasally with microsyringe over the course of 10 min at a 2 min interval. Performed 2x a day for 4 days. Primary neuronal cultures prepared from the cortices of C57BL/6 mice embryos with supplementation of SHED-CM for 24 h. Cell viability determined by WST assay	Passages 3 to 5 after cultured for 48 hours in serum-free DMEM	SHED-CM ameliorates neurological dysfunction in a mouse AD-like model. Mice with SHED-CM exhibited significantly improved RI (recognition index), while rats with BMSC-CM or Fibro-CM exhibited only modestly improved RI. SHED-CM inhibits the generation of 3-NT. Treatment with SHED-CM, BMSC-CM, or Fibro-CM significantly inhibited the generation of both 3-NT & iNOS in AD mice. SHED-CM converts the proinflammatory brain environment of the mouse AD-like model to an anti-inflammatory one & increases neurotrophic factor expression. SHED-CM shifted the M1-type proinflammatory microenvironment associated with mouse AD toward the M2-type anti-inflammatory/neuroprotective one.	SHED-CM provide many neuroreparative effects for the treatment of cognitive deficit; thus, it may provide a novel cell-free neuroreparative therapy for AD.
9	Song et al. 2015	(1) Human dental pulp from healthy permanent teeth (hDPSCs) (2) Human bone marrow mesenchymal stem cells (hMSCs)	14-22 years	Human astrocytes (hAs) Oxygen-glucose deprivation (OGD) model	hDPSCs & hMSCs cultured in serum-free DMEM. CM was collected after 48 h. Pretreatment: hAs were cultured in CM-hDPSCs or CM-hMSCs CM, exposed to oxygen-glucose deprivation (OGD) for 2 h followed by reoxygenation/reperfusion for 30 min. Posttreatment: hAs were cultured in CM-hDPSCs or CM-hMSCs CM, exposed to oxygen-glucose deprivation (OGD) for 2 h followed by reoxygenation/reperfusion for 2 h.	Passages 5 after cultured for 48 hours in serum-free DMEM	hDPSCs and CM-hDPSCs protect against cell death in OGD-induced hAs. Pre- or posttreatment with hDPSCs or CM-hDPSCs conferred a superior cytoprotective effect compared to hMSCs & CM-hMSCs. hDPSCs and CM-hDPSCs inhibit ROS production and IL-1*β* (proinflammatory cytokines) in ischemic hAs. Pre- or posttreatment with hDPSCs or CM-hDPSCs effectively blocked OGD/reperfusion-induced ROS production. Pre- or posttreatment with CM-hDPSCs or CM-hMSCs significantly reduced OGD-stimulated upregulation of IL-1*β*. CM-hDPSCs reduced astrogliosis of ischemic hAs. IF results revealed upregulation of musashi-1 (marker for reactive astrocytes) in OGD-treated hAs. VGF was neuroprotective for RGC. Significantly higher transcription of VGF detected in hDPSC compared to hBMSC/hAMSC.	Pre- & posttreatment with hDPSCs or CM-hDPSCs promote superior cytoprotective effects on hAs, due to reduced gliosis and suppressed free radicals & proinflammatory cytokines.
10	Sugimura-Wakayama et al. 2015 [68]	Human exfoliated deciduous teeth conditioned medium (SHED-CM)	6-12 years	Peripheral nerve injury/Schwann cells/sciatic nerve/human umbilical vein endothelial cells (HUVECs)/human diploid fibroblast/dorsal rat ganglion (DRG)-CNS	SHEDs cultured until 80% confluent & replenished with SF DMEM. CM was collected after 48 h. In vitro: migration-SCs cultured in FBS+DMEM on top chamber of Transwell with supplementation of SHED-CM at the lower chamber for 48 h. Cell removed with swab n filter stained with hematoxylin. Migrated cells were counted. Proliferation-SCs cultured in FBS+DMEM for 24 h. Medium removed & replenished with 100 *μ*l SHED-CM for 48 h. MTT assay was performed. Tube formation assay-mixed HUVECs & HDF seeded under optimal tubule formation conditions. EM medium contains VEGF, HGF, or SHED-CM replaced on days 1, 4, 7, 9, and 11 & incubated with antibody. Total tube lengths & capillary were counted. Neurite length-DRG incubated with SHEM CM and subjected for IF. CCK-8 assay performed to measure cell viability. In vivo: male Wistar/ST rat (250-300 g) left sciatic nerve exposed & isolated. 12 mm nerve segment excised & nerve stumps were pulled 1 mm inside each end of the 12 mm SHEM CM-filled silicon conduit. Rats subjected to walking track analysis, electrophysiological testing, target muscle weight, Masson's trichrome staining & histomorphological analysis.	Unspecified passage number. Conditioned media were collected after 48 hours cultured in serum-free DMEM	SHED-CM enhances SC migration & proliferation. SHED-CM increased SC migration rate by 7-fold & significantly increased proliferation compared to control. Various growth factors present in SHED-CM SHED-CM significantly regulates NTF, angiogenic, & ECM molecules compared to control. SHED-CM stimulates neurite outgrowth & increases DRG neuron viability. Neurite growth & cell viability significantly higher in DRG neuron supplemented SHED-CM compared to control. SHED-CM stimulates angiogenesis *in vitro*. HUVECS cultured in SHED-CM significantly increased tube length & joint number. SHED-CM enhances nerve regeneration. Nerve regenerated in SHED-CM thicker than control. SHED-CM enhances axon regeneration & remyelination. Number of myelinated nerve fiber & degree of myelination are significantly higher in SHED-CM than the other groups. SHED-CM improves motor function recovery. Walking track analysis (SFI) value is significantly higher in SHED-CM compared to control. SHED-CM prevents muscle atrophy & maintains muscle fiber. Gastrocnemius muscle wet weigh & collagen fiber percentage are significantly higher in SHED-CM compared to control.	SHED-CM promotes axonal regeneration & functional recovery in a sciatic nerve defect rat, enhances axon growth, angiogenesis, migration, proliferation, & neuron survival, and thus could be a potential for PNI treatment.
11	Mead et al. 2014	(1) Human dental pulp from healthy permanent teeth (hDPSCs) (2) Human bone marrow mesenchymal stem cells (hMSCs) (3) Human adipose-derived mesenchymal stem cells (hAMSC)	Unspecified age of donor: (1) hDPSCs were purchased from AllCell LLC (Berkeley, CA) (2) hBMSC and hAMSC from Lonza (Slough, UK) (3) Each represented pooled samples from 3 donors	Rat retinal ganglion cells (RGCs)/optic nerve-PNS	DPSCs or BMSCs were cocultured with retinal cells in Transwell chamber system and particular wells were treated 5 *μ*g/ml of Fc-TrKA, Fc-TrKB and/or Fc-TrKC, Fc-VEGFr, Fc-GDNr, Fc-PDGFAr, & Fc-PDGFBr fusion protein inhibitors. Combination of 60 ng/ml NGF, BDNF, & NT-3 added to retinal cells as control. Particular wells of retinal cells treated with 0.1 *μ*m, 1 *μ*m, & 10 *μ*m of VGF.	Passages 2 and 5 after 48 h in serum-free DMEM	hDPSC promoted significantly greater paracrine-mediated neuroprotection and neuritogenesis than hBMSC/hAMSC. hDPSC-treated retinal cultures showed significant RGC survival than that in hAMSC but not significantly than hBMSC. NTFR Fc-receptor blockers for multiple NTFR attenuated the neuroprotective and neuritogenic effect of hDPSC/hBMSC/hAMSC. NTFR blockers significantly attenuated hDPSC-mediated neuroprotection or neuritogenesis of cocultured RGC compared to uninhibited hDPSC/retinal cell cocultures. hDPSC, hBMSC, and hAMSC have distinct NTF expression profiles. Both NTF genes showed distinct expression in each stem cells. hDPSC secrete multiple NTF at higher levels than hBMSC/hAMSC. hDPSC secreted significantly greater titers of NTF factors than hBMSC/hAMSC.	hDPSC mediates neuroprotection & neuritogenesis through paracrine effects of secreted neutrophic factors; thus, hDPSCs may represent an effective cellular therapy for nerve repair.
12	Ishizaka et al. 2013	Porcine premolar teeth (CD31)-side population (SP) cells: (1) Porcine dental pulp (2) Porcine bone marrow (3) Porcine adipose	Unspecified	(1) NIH3T3 mouse embryonic fibroblast (2) Human peripheral blood mononuclear cells (PBMCs) (3) Human neuroblastoma cell line TGW	CM from DP, BM, & AD collected after 48 h of culture & concentrated using filter unit. In vitro: MEF: MEF cultured in DMEM+FBS for 24 h, then changed into DMEM+each CM (final conc. of 5 *μ*g/ml). Cells were subjected to cell count & migration Apoptosis-MEF grown in DMEM (3 days) & incubated with staurosporine (100 nm) supplemented 5 *μ*g/ml of each CM. After 8 h, MEF was analyzed by flow cytometry. Endothelial cell differentiation-MEF cultured in EBM2 + 2%FBS & 5 *μ*g/ml of each CM & performed ICC. MEF seeded on Matrigel in EBM2 + 2%FBS, 5 mg/ml heparin, 5 mg/ml ascorbic acid, 5 mg/ml hydrocortisone, & 5 mg/ml of each CM. Network formation observed after 4 h. Lengths of networks of cords & tube-like structures was measured. PBMCs: immunomodulatory effect-cells were purified & treated with mitomycin C (3 h). Autologous PBMCs & allogenic stimulator PBMCs were cocultured & supplemented with 5 *μ*g/ml of CM. Cell counted at 0, 12, 24, & 36 h. Human neuroblastoma cell line TGW: neurite outgrowth-TGW were serum starved & stimulated with each CM (48 h). Neurite length was measured. 100 cells/sample were counted.	Unspecified passage number. Conditioned media were collected after 48 hours cultured in serum-free DMEM	CM from DP produced higher migration activity, antiapoptotic activity, & immunomodulatory effects compared to BM & AD. Stimulatory effects of the CM from BM on migration & immunosuppression significantly higher than CM from AD. HUVECs differentiated into endothelial cells that were positive for VE-cadherin & formed extensive networks of cords and tube-like structures as early as 4 h with supplementation of each CM. CM from DPs had higher angiogenic potential than CM from AD. CM from DP had significantly higher stimulatory effects on neurite outgrowth than CM from BM & AD.	CM from DP had higher trophic effects on angiogenesis, neurite outgrowth, migration, antiapoptosis, & immunomodulation than BM & AD CM in vitro.
13	Mead et al. 2013	Upper and lower incisors of Sprague-Dawley rats weighing 170 to 200 g: (1) Rat dental pulp stem cells (DPSCs) (2) Rat bone marrow mesenchymal stem cells (BMSCs)	Unspecified	Rat retinal ganglion cells (RGCs)/optic nerve-PNS	Rats (weighing 170-200 g): *In vitro*: DPSCs or BMSCs-CM was collected after 48 h of culture & subjected for neurotrophins quantitation using ELISA. DPSCs or BMSCs were cocultured with retinal cells in Transwell chamber system and particular wells were treated with protein inhibitors. CM pooled from 3 samples for coculture & transplant. *In vivo*: intraorbital optic nerve crush (ONC) was exposed & crushed using forceps 1 mm posterior to the lamina cribrosa. ONC was created in rats' eye and injected intravitreally (150,000 cells suspended in 5 ml PBS) either with DPSC, BMSC, or PBS (control). Every 7 days, optical coherence tomography (OCT) was performed to measure retinal nerve fiber layer (RNFL).	Passages 2 to 4 after cultured for 48 hours in serum-free DMEM	DPSCs secreted NGF, BDNF, & NT-3. These neurotrophic titers were higher in DPSCs-CM compared to BMSCs-CM. DPSCs promoted BIII-tubulin retinal cell survival & neurogenesis in a coculture assay. DPSCs promote significant increase in the survival & number of cocultured retinal cells compared with retinal cultured alone, cocultured with BMSCs or treated with recombinant factors. Fc-TrK receptor attenuates survival & neuritogenesis effects of DPSCs. DPSCs cocultured significantly decreased number of BIII-tubulin retinal cells after treated with Fc-TrKA, Fc-TrKB, & Fc-TrKC, whereas BMSCs cocultured reduced retinal cells survival in Fc-TrKA, Fc-TrKB, or combination of 3, but not after adding Fc-TrKC alone. DPSC transplants preserved RNFL thickness for 14 days after ONC injury. All animal survived with no adverse effects. There were no significant RNFL thinning at 7 pdl that injected with living DPSC compared to intact animals, indicating a neuroprotective effect of DPSC. Transplanted DPSCs survived for 21 days. Viable DPSCs detected in the transplanted site at 21 pdl with higher level of BDNF & NT-3 compared to eyes transplanted with dead DPSCs. DPSC transplant protects RGCs from death after ONC. DPSC transplant after ONC significantly increased RGC survival at 21 dl compared with animals received BMSCs, dead DPSCs, or ONC alone. DPSC transplant after ONC promotes RGC axon regeneration. At distances 100-1200 *μ*m distal to crush site, number of regenerating GAP-43 RGC axon significantly increased in DPSCs compared with BMSCs, dead DPSCs, or untreated.	DPSCs secrete higher concentration of neurotrophins that are responsible for promoting axotomized RGC neuroprotection and neuritogenesis/axogenesis; hence, it may be a promising alternative for CNS cell therapy.
